# A polymeric diet rich in transforming growth factor beta 2 does not reduce inflammation in chronic 2,4,6-trinitrobenzene sulfonic acid colitis in pre-pubertal rats

**DOI:** 10.1186/s12876-020-01574-8

**Published:** 2020-12-10

**Authors:** Claire Dupont-Lucas, Rachel Marion-Letellier, Mathilde Pala, Charlène Guerin, Asma Amamou, Marine Jarbeau, Christine Bôle-Feysot, Lionel Nicol, Amelyne David, Moutaz Aziz, Elodie Colasse, Céline Savoye-Collet, Guillaume Savoye

**Affiliations:** 1 INSERM UMR 1073, Institute for Biomedical Research, Rouen University, Rouen, France; 2 Department of Pediatrics, Caen University Hospital, Caen, France; 3 INSERM UMR 1096, Institute for Biomedical Research, Rouen University, Rouen, France; 4 Department of Pathology, Rouen University Hospital, Rouen, France; 5 Department of Nutrition, Rouen University Hospital, Rouen, France; 6 Department of Radiology, Rouen University Hospital, Rouen, France; 7 QUANTIF-LITIS EA 4108, Rouen University, Rouen, France; 8 Department of Gastroenterology, Rouen University Hospital, Rouen, France

**Keywords:** Transforming growth factor beta 2, Fibrosis, Inflammation, Growth and development

## Abstract

**Background:**

Pediatric Crohn’s disease is characterized by a higher incidence of complicated phenotypes. Murine models help to better understand the dynamic process of intestinal fibrosis and test therapeutic interventions. Pre-pubertal models are lacking. We aimed to adapt a model of chronic colitis to pre-pubertal rats and test if a polymeric diet rich in TGF-β2 could reduce TNBS-induced intestinal inflammation and fibrosis.

**Methods:**

Colitis was induced in 20 five-week-old Sprague–Dawley male rats by weekly rectal injections of increasing doses of TNBS (90 mg/kg, 140 mg/kg and 180 mg/kg) for 3 weeks, while 10 controls received phosphate-buffered saline. Rats were anesthetized using ketamine and chlorpromazine. After first administration of TNBS, 10 rats were fed exclusively MODULEN IBD® powder, while remaining rats were fed breeding chow. Colitis was assessed one week after last dose of TNBS by histopathology and magnetic resonance colonography (MRC).

**Results:**

Histological inflammation and fibrosis scores were higher in TNBS group than controls (*p* < 0.05 for both). MRC showed increased colon wall thickness in TNBS group compared to controls (*p* < 0.01), and increased prevalence of strictures and target sign (*p* < 0.05). Colon expression of COL1A1, CTGF, α-SMA and COX-2 did not differ between TNBS rats and controls. TNBS colitis was not associated with growth failure. Treatment with MODULEN IBD® was associated with growth failure, increased colon weight/length ratio (*p* < 0.01), but did not affect histological scores or MRI characteristics. Colon expression of α-SMA was significantly lower in the MODULEN group versus controls (*p* = 0.005).

**Conclusion:**

Features of chronic colitis were confirmed in this model, based on MRC and histopathology. Treatment with MODULEN did not reverse inflammation or fibrosis.

## Background

Crohn’s disease is a chronic inflammatory bowel disease (IBD) characterized by flares and periods of remission, leading to progressive tissue damage. Complicated phenotypes of disease include fistulizing and stricturing bowel disease. Bowel stenosis, defined as luminal narrowing with obstructive symptoms or pre-stenotic dilation on imaging, occurs in 20 to 40% of patients during the first ten years of disease and is the main cause for surgery in IBD [1]. Pediatric-onset IBD has a more severe phenotype than adult IBD with a higher prevalence of stricturing and penetrating disease [2, 3]. In addition, Crohn’s disease beginning before puberty can lead to significant growth failure and delayed puberty. Ongoing efforts are made to reverse the complex inflammatory process, and drugs targeting multiple steps of the inflammatory process have been developed. Intestinal fibrosis occurs in the context of inflammation leading to tissue damage and altered tissue reconstruction with excessive extracellular matrix deposition [4]. Intestinal fibrosis is a dynamic process, and mechanisms of initiation and perpetuation of the process are not yet fully understood. Evidence from other diseases tends to show reversibility of tissue fibrosis (i.e. liver, kidney or lung fibrosis). However, to date, there is no effective treatment for reversing intestinal fibrosis. Efforts are thus made to prevent the fibrotic process through several targets [5]. In order to test the drugs, murine models have been developed. Since fibrosis is a dynamic procedure, non-invasive techniques are needed to follow progression of fibrosis. Our team has previously described a model of chronic colitis in adult rats that had specific characteristics on MR-colonography [6]. To our knowledge this has never been done in pre-pubertal animals. Based on the hypothesis that colitis might develop more quickly and severely in pre-pubertal rats as in humans, the primary aim of this study was to adapt the model of chronic colitis to young pre-pubertal rats and confirm the colitis on MR-colonography.

Enteral nutrition with a polymeric diet given exclusively for 6 to 8 weeks is the recommended treatment for induction of remission of Crohn’s disease in children with mild to severe disease, according to the European Society for Pediatric Gastroenterology, Hepatology and Nutrition (ESPGHAN) and the European Crohn’s and Colitis Organization [7]. In particular, MODULEN IBD®, a polymeric diet rich in TGF-β2 has shown rates of clinical remission of up to 79% in children [8]. Several teams have shown promising results with MODULEN IBD® as maintenance therapy in pediatric Crohn’s disease. MODULEN IBD supplementation has been shown to reduce clinical colitis scores in chronic DSS colitis in adult mice [9], however there have been no studies on the anti-inflammatory effects of MODULEN in pre-pubertal animal models. In addition, although several molecules targeting the mechanisms of intestinal fibrosis have been tested [5], there is a lack of studies on the effect of nutrition, and in particular MODULEN, on intestinal fibrosis. The secondary aim of our study was thus to evaluate if a maintenance treatment with MODULEN IBD® in a pre-pubertal rat model of chronic colitis could have an effect on inflammation and/or prevent fibrosis.

## Methods

### Study design

**Fig. 1:**
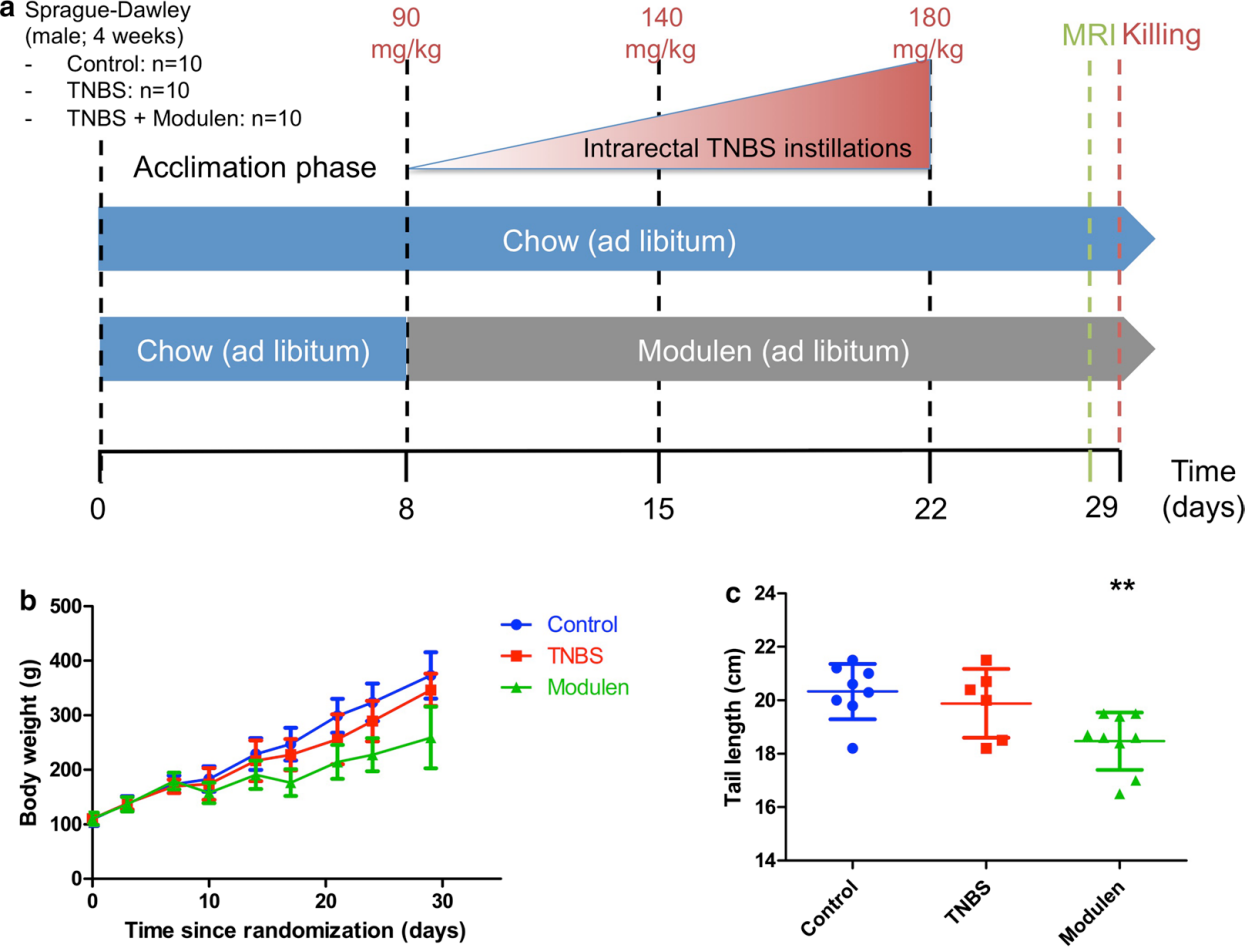
Experimental protocol and growth. **a** Experimental protocol for induction of chronic colitis in pre-pubertal rats, and treatment with MODULEN IBD®. **b** Rat body weight by group (**p < 0.01 for between group effect and time effect, 2-group comparison, mixed model). **c** Tail length at the end of the study, by group (**p < 0.01 vs. controls, 2-tailed). For all panels, Control group: n = 10, TNBS group: n = 10, TNBS+ Modulen group: n = 10

Thirty 4-week-old Sprague Dawley male rats were purchased from Janvier labs (Le Genest Saint Isle, France). After one week of acclimatization they were randomly allocated to one of three study groups: Control (n = 10), TNBS (n = 10) and TNBS+MODULEN (n = 10) (Fig. 1, Panel a). The rats were housed 5 per standard cage to provide for their interaction needs, were exposed to light/dark cycles of 12 h each and provided with water ad libitum. Starting from first administration of TNBS, the TNBS+MODULEN group received MODULEN IBD® powder as sole food source. The other groups received a standard rat breeding diet (A03, SAFE) in a powdered form. All sections of this report adhere to the ARRIVE Guidelines for reporting animal research. A completed ARRIVE guidelines checklist is included in Checklist S1. All animals were included in the study; there were no exclusion criteria established a priori. Sample size was calculated using G*Power Software. Based on our previous study [6], a difference in colon wall thickness on MRC between controls and TNBS group was shown with an effect size of 3.65, and mean mortality rate was 25%. We estimated that the effect size would be 50% lower using a number of doses of TNBS reduced by 50% to take into account higher susceptibility to drugs in young animals. To achieve power = 0.90 and alpha = 0.05 to detect this difference would require 8 animals per group, which we increased to 10 based on a predicted attrition rate of 25%.

### Induction of colitis

From the age of 5 weeks, colitis was induced by weekly intra-rectal instillation of 2,4,6-trinitrobenzene sulfonic acid (TNBS, Sigma-Aldrich, Saint-Quentin Fallavier, France) for 3 weeks. After fasting for 24 h, rats were anesthetized by intra-peritoneal injection of ketamine 8 mg/kg (Panpharma) and Chlorpromazine 1 mg/kg (Sanofi-Aventis). A polyurethane cannula was inserted 3 cm into the rectum and TNBS—ethanol was injected, at weekly increasing doses of 90, 140 and 180 mg/kg of body weight. TNBS was dissolved in a 50% Ethanol vehicle, for a total volume of 250 μL. The rats were maintained in a head-down position for 5 min after the injection to minimize leaks. Rats in the control group received an intra-rectal instillation of 250 μL of phosphate-buffered saline (PBS). The rats were selected in random order from one of the 6 cages (controls, TNBS and MOD groups) and received the treatment (TNBS or PBS) depending on their allocation group. During the study protocol, rats were weighed twice a week and observed for signs of pain or significant weight loss.

### Magnetic resonance colonography

One week after the third intra-rectal injection, a magnetic resonance colonography was performed. Rats were anesthetized by intra-peritoneal injection of Thiopental (Panpharma) at the dose of 90 mg/kg. Heart rate was monitored during the MRI by surface electrodes. Rats were placed in the cradle in a supine position. A tube of circulating warm water around the rat during MRI and a warming plate during recovery phase prevented hypothermia. The rats were selected in random order from one of the 6 cages (controls, TNB, MOD) in order to minimize potential confounders such as time since induction of colitis and cage location.

MRI was performed with a small animal machine: Bruker BioSpec 47/40USR, 4.7 T (Bruker Biospin, Ettlingen, Germany). There was no injection of antispasmodic or contrast agent. Respiratory movements were corrected using the Intragate™ technique. Four sequences were performed; the total acquisition time was 42 min per animal. Parameters for the T2-weighted (T2w) RARE (rapid acquisition with relaxation enhancement) sequence were: TR 5443 ms, TE 34 ms, matrix 320 × 224, slice 1 mm, NEX 3, flip angle 180°, field of view (FOV) 5.0 × 5.3 cm, acquisition time: 10 min, RARE factor 8. Parameters for T2-w RARE with fat suppression (FAT-SAT): TE 39 ms, TR 6027 ms, RARE factor 8, NEX 3, acquisition time 8 min, flip angle 180°, slice 1 mm, FOV 5.0 × 5.3 cm, matrix 320 × 224. T1-weighted sequence with intragate FLASH (fast low angle shot): TR 413 ms, TE 2.8 ms, matrix 256 × 256, slice 1.1 mm, flip angle 80°, FOV 4.5 × 4.5 cm; acquisition time: 14 min. Diffusion sequence: TE 2.6 ms, TR 5.2 ms, number of segments 44, NEX 1, flip angle 50°, slice 1 mm, FOV 4 cm, matrix 128 × 128, acquisition time 10 min, b values: 0 s/mm^2^, 100 s/mm^2^, 200 s/mm^2^.

MRI images were analyzed in DICOM, using the ParaVision 5.0 software. A senior radiologist (C. S.-C.) who was blinded to the allocation group interpreted all images.

Image quality taking into account respiratory and bowel wall movements was assessed on a scale of 0 (poor quality) to 3 (excellent quality). Measures were made in the descending colon.

In order to assess inflammation, the criteria used, similarly to our previous work [6] were: maximal colon wall thickness (average of 3 measures), minimal colon wall thickness (average of 3 measures), colon wall thickness at splenic angle (average of 3 measures), colon wall signal intensity in Regions of Interest (ROI) on T2w sequences (average of 2 measures), target sign (present or absent), ulcerations, spontaneous enhancement of colon wall signal on T1w sequences. Signal intensity in ROI was measured in the descending part of the colon in axial plane, which anatomically allows a cross section of the bowel. Size of ROI was adapted to the wall thickness: 0.0002 cm^2^. The results presented are the mean value of measurements performed.

The MRI signs suggesting fibrosis, according to our previous work [6], were: narrowing of the colon lumen ((maximal diameter − minimal diameter) × 100/maximal diameter), a stricture defined as narrowing of the colon lumen associated with proximal colon dilation, and the presence of a mucosal flap in the lumen.

### Euthanasia and samples

After completion of the MR-colonography, rats were euthanized by a lethal dose of intraperitoneal thiopental and then decapitated. Tail was measured as a proxy for growth. Colon was resected, washed with PBS to remove feces, measured and weighed. Six one-centimeter samples were taken from the colon, starting from the rectum, one of which was fixed in 10% neutral-buffered formalin (Sigma-Aldrich) for histology, the others stored at − 80 °C for Western-Blot and PCR.

### Histology

Histological analyses were performed by a single pathologist (M.A.), blinded to allocation group, on a sample of distal colon taken 4 cm from the anus. The formalin-fixed samples were embedded in paraffin, and 5-µm sections were colored with hematoxylin and eosin (H&E; Merck, Darmstadt, Germany) for standard histology. Samples were studied on 3 levels of cut.

Inflammation was scored using a semi-quantitative score previously used by our team: from 0 (no inflammation) to 3 (severe inflammation) [6]. Fibrosis was scored from 0 (no fibrosis) to 3 (severe fibrosis). Images were taken by standard light microscopy using a Leica microscope.

### Colon expression of cyclooxygenase-2 (COX-2) and alpha smooth muscle actin (α-SMA) by Western Blot

Western Blot of COX-2 and α-SMA was performed as previously [6].

### Alpha-1 type I collagen (COL1A1), connective tissue growth factor (CTGF) and alpha smooth muscle actin (α-SMA) mRNA

Colon samples were frozen in liquid nitrogen and stored at − 80 °C before RNA preparation. Total RNA was isolated using the guanidium isothiocyanate method and reverse transcribed into cDNA. Colon mRNA expression of COL1A1 (primer sequences F:ACTCAGCCCTCTGTGCCT, R: CCTTCGCTTCCATACTCG), CTGF (F: GGCAGGGCCAACCACTGTGC, R: CAGTGCACTTGCCTGGATGG), α-SMA (F:CTTCTATAACGAGCTTCGC, R: TCCAGAGTCCAGCACAAT) and the internal control (GAPDH) was measured by qPCR as previously [10].

### Statistics

Distribution of variables was described by mean ± SD for normally distributed quantitative variables, and percentage for qualitative variables. We used non-parametric Wilcoxon rank sum tests for 2-group comparison with Wilcoxon exact test correction for small numbers and Fisher’s exact test for qualitative variables. Growth curves were compared between groups using a mixed model studying the effects of time, group and time*group. Analyses were replicated using an Analysis of Variance for repeated measures. Statistical analyses were performed using SAS 9.2 and Graph Pad Prism 5. All tests were two-tailed and a *p *value < 0.05 was considered significant. Rats that died before the study endpoint could not be submitted to MR-colonography and their colons could not be sampled for protein or gene expression. These rats were not included in the final analyses.

### Ethical considerations

Animal care and experimentation complied with the European directive for the use and care of laboratory animals (Directive 2010/63/UE) and received approval from the Institutional Care and Use Committee (Comité d’Ethique Normande en Matière d’Expérimentation Animale, CENOMEXA- registration number: APAFIS#16184-2018071711054339). Rachel Marion-Letellier is authorized by the French Government to use the present rat model (Authorization no. 76-106). Animal welfare was monitored daily by visual inspection and all interventions were done during the light cycle. Painful procedures were carried out under general anesthesia and all efforts were made to minimize suffering.

## Results

### Survival and growth

Growth curves of TNBS rats and controls did not differ during the protocol, and final weight and tail length were not significantly different between the TNBS group and the control group: 346.7 ± 29.7 g versus 373.1 ± 42.5 g and 19.8 ± 1.3 cm versus 20.3 ± 1.0 cm respectively (Fig. 1, Panel b, c). The survival rate was 50% (5/10) in the TNBS group: 3 rats died at TNBS injections n°1 and 2, 1 rat had to be euthanized due to intestinal occlusion after 2nd dose of TNBS, 1 died during anesthesia for MRI but the colon could be analyzed for histology. In the control group, 2 rats died at PBS injection n°2, the overall survival rate in the control group was 80%.

### Validation of the chronic colitis model: histology

**Fig. 2:**
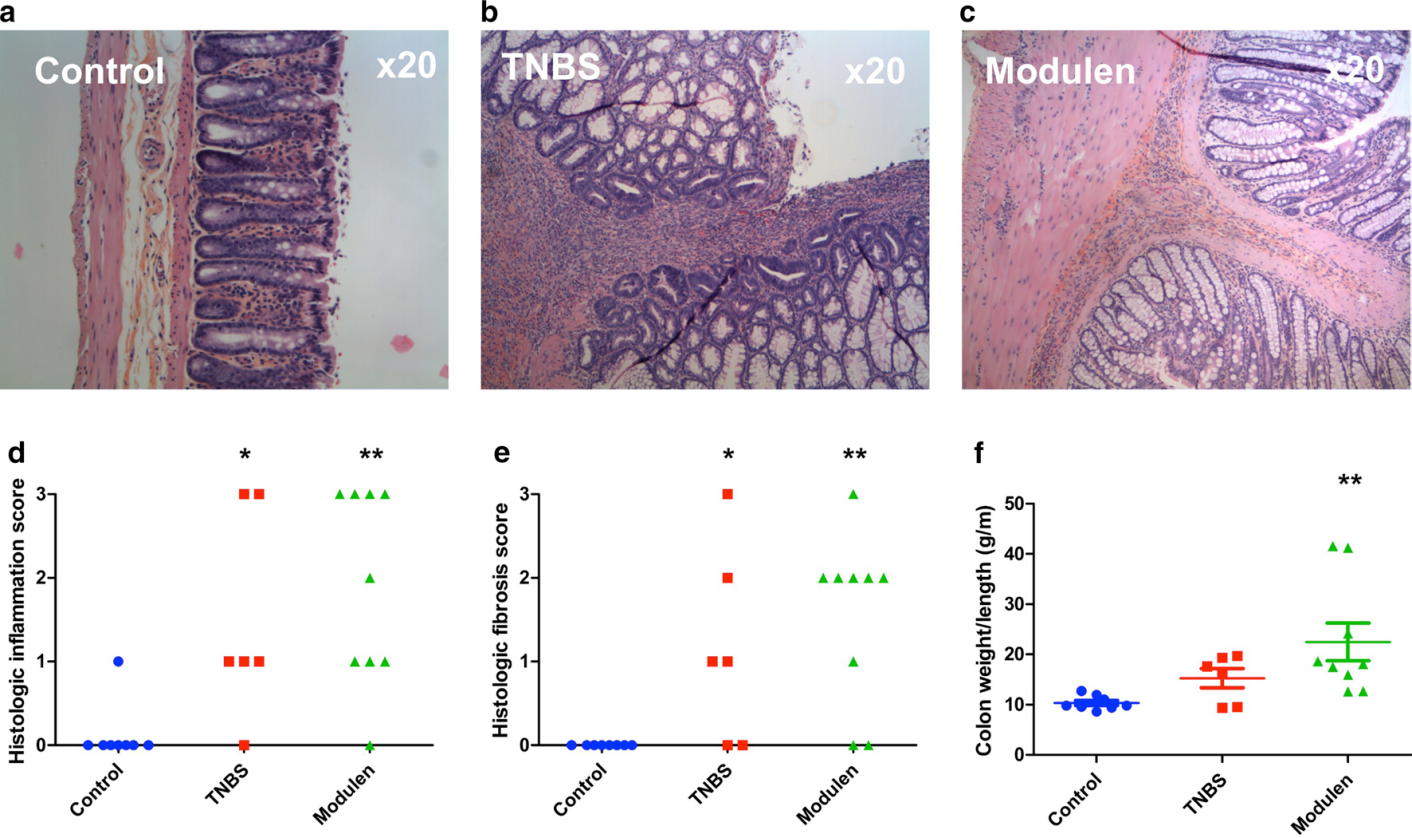
Validation of the pre-pubertal chronic colitis model: histology. **a** Representative images of H&E from the Control group showing normal histology. **b** Representative images of H&E from the TNBS group showing severe transmural inflammation with glandular regeneration and severe fibrosis. **c** Representative images of H&E from the TNBS+MODULEN group showing severe inflammation and severe fibrosis. **d** Histological inflammation score by group. **e** Histological fibrosis score by group. **f** Colon weight/length ratio by group. *p < 0.05, **p < 0.01, significant comparisons are versus Control group unless otherwise specified. Panels **d**–**f** Control group: n = 8, TNBS group: n = 6, TNBS+Modulen group: n = 9

Colon weight/length ratio (g/m) did not differ significantly between the TNBS and the control group. However, colon length was shorter in the TNBS group than in the control group (*p* = 0.02) (Fig. 2, Panel f).

Eighty-three per cent (5/6) of rats from the TNBS group had mild to severe inflammation based on H&E histology, compared to 13% (1/8) in the control group (*p* = 0.04) (Fig. 2, Panel d). Sixty-seven per cent (4/6) of rats in the TNBS group had mild to severe fibrosis based on histological score, compared to none in the control group (*p* = 0.015) (Fig. 2, Panel e).

### MR-colonography characteristics of the TNBS chronic colitis

**Fig. 3:**
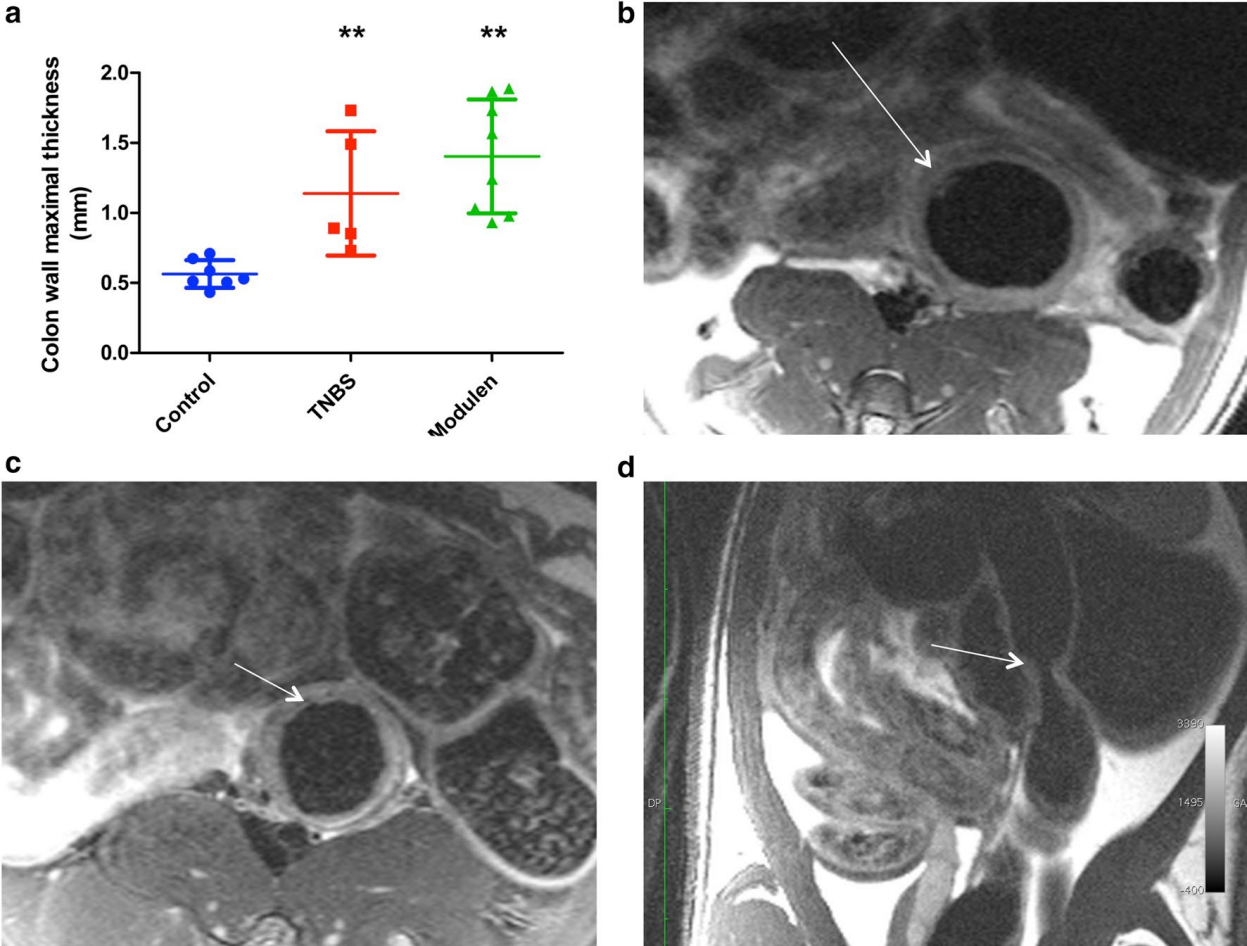
Validation of the pre-pubertal chronic colitis model: MRI-colonography. **a** Colon wall thickening: maximum thickness by group (**p < 0.01 vs. controls, 2-tailed). Control group: n = 7, TNBS group: n = 5, TNBS+Modulen group: n = 8. **b** MRI axial plane T2w image of the descending colon, TNBS group. The arrow indicates a Target sign. **c** MRI T2w image of the descending colon, TNBS+MODULEN group. The arrow indicates irregularities of the colon wall suggestive of an ulcer, with increased T2w signal intensity. **d** MRI image of the descending colon, TNBS group. The arrow indicates colon stenosis, without T2w hypersignal, which could suggest a fibrotic stricture

Image quality was deemed satisfactory for analyses, although wall artifacts occurred in 5/7 rats in the control group. Colon wall thickness in T2-weighted images in the axial plane was significantly higher in the TNBS group than in the control group, with a mean maximal thickness of 1.14 ± 0.44 mm in the TNBS group versus 0.56 ± 0.10 mm in the control group (*p* = 0.003) (Table 1, Fig. 3, Panel a). Colon stenosis was observed in 60% of rats in the TNBS group versus none of the controls (*p* = 0.046) (Fig. 3, Panel d). A target sign was observed in 60% of rats in the TNBS group versus none of the controls (*p* = 0.046) (Fig. 3, Panel b). There was no difference between TNBS and control groups regarding increased wall signal intensity on T2w sequences, spontaneous enhancement of T1w signal intensity or colon wall ulcers. Diffusion analyses could not be interpreted due to excessive noise in proportion to wall thickness.

**Table 1:** MR-colonography characteristics, by study group

	Control (n = 7)	TNBS (n = 5)	MODULEN (n = 8)	TNBS/CTL (*p*)	TNBS/MOD (*p*)	MOD/CTL (*p*)
*Colon wall thickness (axial T2)*						
Maximum thickness (mm)	0.56 ± 0.10	1.14 ± 0.44	1.40 ± 0.41	0.003	NS	0.0003
Minimal thickness (mm)	0.50 ± 0.07	0.97 ± 0.23	1.09 ± 0.24	0.003	NS	0.0003
Thickness at kidney hilum level (mm)	0.54 ± 0.07	1.03 ± 0.34	1.07 ± 0.16	0.003	NS	0.0003
Stenosis (n, %)	0 (0)	3 (0.60)	4 (0.50)	0.046	NS	0.08
Mucosal flap (n, %)	0 (0)	2 (0.40)	7 (0.88)	NS	NS	0.001
Increased wall signal intensity on T1w (n, %)	0 (0)	1 (0.20)	2 (0.25)	NS	NS	NS
Irregular patterns of mucosal wall (ulcers) (n, %)	0 (0)	1 (0.20)	5 (0.63)	NS	NS	0.03
T2w wall intensity (mean of 2 ROI measures)	1303.7 ± 375.3	1413 ± 208	3210 ± 2531	NS	NS	NS
Increased wall signal intensity on T2w (n, %)	0 (0)	2 (0.40)	4 (0.50)	NS	NS	0.08
Target sign—yes (n, %)	0 (0)	3 (0.60)	3 (0.38)	0.046	NS	NS

TNBS-induced chronic colitis in pre-pubertal rats. Groups were compared 2 by 2 using non-parametric Wilcoxon rank sum tests for 2-group comparison with Wilcoxon exact test correction for small numbers and Fisher’s exact test for qualitative variables. All tests were two-tailed and a *p *value < 0.05 was considered significant

*ROI* region of interest

### Biological markers of inflammation and fibrosis

**Fig. 4:**
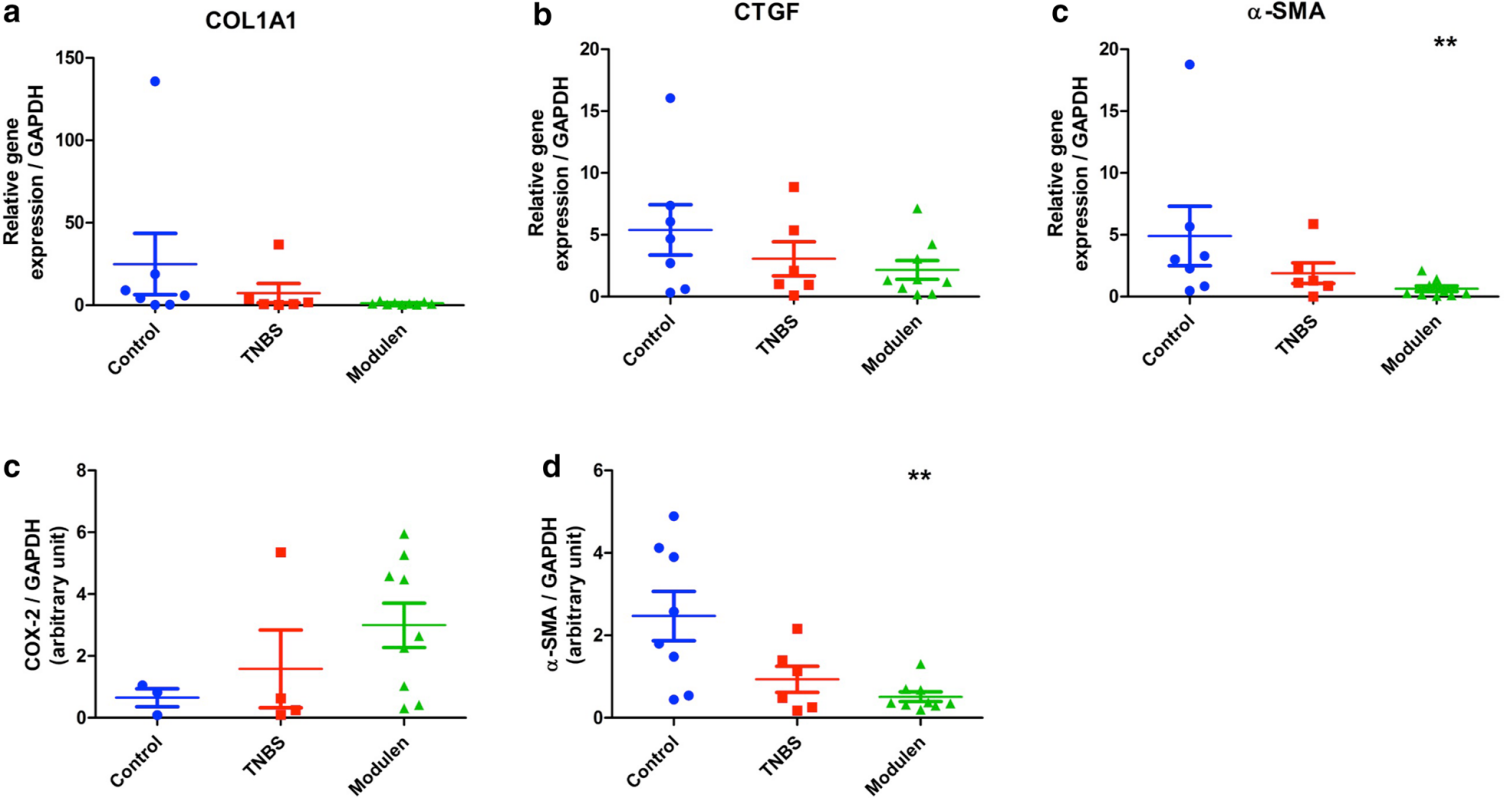
Expression of inflammatory markers in colonic tissue homogenates, TNBS-induced chronic colitis model. Gene expression of COL1A1 (Panel **a**), CTGF (Panel **b**) and α-SMA (Panel **c**) measured by RT-PCR (**p < 0.01 vs. controls, 2-tailed); Protein expression of COX-2 (Panel **d**) and α-SMA (Panel **e**) measured by Western Blot (**p < 0.01 vs. controls, 2-tailed). Sample size by panel: Panel **a**–**c** Control n = 7, TNBS n = 6, TNBS + Modulen n = 9. Panel **d** Control n = 3, TNBS n = 4, TNBS + Modulen n = 9. Panel **e** Control n = 8, TNBS n = 6, TNBS + Modulen n = 9

There was no difference in gene expression levels of COL1A1, CTGF and α-SMA relative to GAPDH, between controls and TNBS group. Mean level of COL1A1 was 7.30 ± 5.91 in the TNBS group versus 24.92 ± 18.63 in the control group (NS) (Fig. 4, Panel a). CTGF was 3.05 ± 1.38 in TNBS group versus 5.39 ± 2.03 in the control group (NS) (Fig. 4, Panel b). α-SMA was 1.90 ± 0.85 in the TNBS group versus 4.91 ± 2.40 in the control group (NS) (Fig. 4, Panel c). Protein expression of α-SMA and an inflammatory marker, COX-2, measured by Western Blot did not differ between TNBS and control group (Fig. 4, Panel d, e).

### Effect of MODULEN IBD® in a model of chronic colitis in pre-pubertal rats

#### Survival and growth

The body weight growth curve of rats in the TNBS+MODULEN group was lower than those of the other groups (*p* < 0.0001 mixed model), and final weight and tail length were significantly lower in the TNBS+MODULEN group than in the TNBS group: 259.1 ± 56.4 g versus 346.7 ± 29.7 g (*p* = 0.004) and 18.5 ± 1.1 cm vs 19.9 ± 1.3 cm (*p* = 0.04) (Fig. 1, Panel b, c). Final body weight and tail length were also lower than control group (*p* = 0.0003 and *p* = 0.003 respectively). Rats in the TNBS+MODULEN group survived colitis induction and sedation procedures better: only one rat died prematurely in the TNBS+MODULEN group, in the 24 h following the 3rd dose of TNBS.

### Effect of MODULEN IBD® on TNBS chronic colitis based on histopathology

Colon weight/length ratio (g/m) did not differ significantly between the TNBS+MODULEN group and the TNBS group: 22.5 ± 11.2 versus 15.3 ± 4.7 g/m (*p* = NS), but was significantly higher than the control group (*p* = 0.0002) (Fig. 2, Panel f).

Eighty-nine per cent (8/9) of rats from the TNBS+MODULEN group had mild to severe inflammation based on histology, compared to 83% (5/6) in the TNBS group (*p* = NS), and compared to 13% (1/8) in the control group (*p* = 0.006) (Fig. 2, Panel d).

Seventy-eight percent (7/9) of rats from the TNBS+MODULEN group had mild to severe fibrosis based on histology, compared to 67% (4/6) of rats in the TNBS group (*p* = NS) and none of the controls (*p* = 0.006) (Fig. 2, Panel e).

### Effect of MODULEN IBD® on TNBS colitis based on MR-colonography

MRI characteristics of rats from the TNBS+MODULEN group did not differ from those of the TNBS group for any of the studied characteristics (Table 1). When compared to the control group, rats from the TNBS+MODULEN group had an increased colon wall thickness (*p* = 0.0003), and developed more frequently a mucosal flap (*p* = 0.001) or ulcers (*p* = 0.03).

### Effect of MODULEN IBD® on TNBS colitis based on biological markers

Colon COL1A1, CTGF and α-SMA mRNA levels did not differ between TNBS+MODULEN group and TNBS group (Fig. 4, Panel a–c). When compared to controls, the rats in the TNBS+MODULEN group had a lower α-SMA gene expression (*p* = 0.005). This result was confirmed by protein expression of α-SMA by Western Blot (*p* = 0.003 compared to controls). Colon expression of COX-2 did not differ between the 3 groups (Fig. 4, Panel e).

## Discussion

In this study we have shown that a protocol of 3 intra-rectal TNBS injections successfully causes colitis in pre-pubertal rats, characterized by inflammation and fibrosis on histology, and MR-colonography, although the rats did not exhibit growth delay.

The TNBS chronic colitis model in mice developed by Lawrance et al*.* showed interesting properties mimicking natural history of IBD in humans, with a progressive reduction in the proportion of mice having severe inflammation between the 2nd and 6th dose of TNBS, and a progressive increase of the incidence of severe fibrosis (50% of mice at the 6th dose) [11]. Lawrance et al*.* also showed the dynamic nature of fibrosis, persisting 2–4 weeks after the last dose of TNBS.

There have been very few published studies on chronic TNBS colitis in pre-pubertal rats [12, 13]. Hence our first objective was to adapt the TNBS chronic colitis model to young rats, with low mortality and significant fibrosis before reaching adult age. The window of opportunity was narrow, since in Sprague–Dawley rats the peri-natal period lasts until Postnatal Day 21 (PND 21), the pre-pubertal period until PND 42 (6 weeks) and the pubertal period until PND 63 (9 weeks) [14]. We chose to induce colitis starting at 5 weeks of age and all rats were analyzed at 8 weeks of age, before the end of the pubertal period.

Among the signs of colitis expected in a TNBS model, weight loss and linear growth failure are classical features [12, 13]. In our model there was no significant growth failure based on weight and tail length in the TNBS group, although significant histological inflammation indicates that the dose of TNBS was sufficient. A possible explanation results from the fact that the rats were fed a high energy high protein (25.2% crude protein) rat breeding diet during the whole study instead of transitioning to normal diet as they increased in age, in order to allow between group comparisons with the MODULEN IBD® fed rats. In comparison, rats in other chronic TNBS models are fed a standard rat chow with normal protein intake (generally 17–19% crude protein). In this case, the rat breeding diet might have had the effect of nutritional supplementation that could have counterbalanced the detrimental nutritional effect of colitis. Ballinger et al*.* showed, in a model of acute TNBS colitis in pre-pubertal Wistar rats with pair fed controls that colitis caused impaired linear growth caused in part by inflammation and in part by undernutrition [13]. Nutritional supplementation in the colitis group increased weight almost to the value of controls [13].

An other possible explanation for the absence of growth failure in the TNBS group could be that the rats with the most severe colitis died during the protocol and that those that survived had a better nutritional status. Indeed there was a significant mortality rate of 5/10 in the TNBS group versus 2/10 in the control group. We adapted and lowered the doses of TNBS compared to those previously reported, but maybe insufficiently for these young animals. In the absence of comparable studies in this age group it might be necessary in future studies to lower the doses 2 and 3 of the protocol.

Choosing the correct dose of TNBS is a challenge when putting into balance colonic inflammation, fibrosis and morbidity. Among the animal models of intestinal fibrosis [15], the chronic TNBS colitis requires repeated doses of TNBS to induce fibrosis. The effect of TNBS also has a high variability between species and between animals in an experimental group [11, 16]. Previous studies have shown that TNBS models based on 2–3 doses of TNBS cause severe but transient colitis. Fibrosis, as confirmed by elevated expression of collagen and components of the extra-cellular matrix, becomes particularly evident after 4 doses [12], 6 doses [6] and even 8 [11] repeated administrations of TNBS.

Our study is the first to assess colitis in pre-pubertal rats using MR-colonography. The MR-colonography characteristics of our model were similar to our previously reported adult rat chronic TNBS colitis model, with a significant increase of colon wall thickness and stenosis with no spontaneous enhancement of T1w wall signal [6]. There is persisting need for imaging techniques that could quantify the amount of fibrotic tissue in strictures and assist the decision of medical or surgical management. There have been advances in assessment of fibrostenotic Crohn’s disease using cross-sectional imaging techniques [17]. Diffusion MR techniques are promising, but could not be applied to our model due to the small size of the animal colons and the “noise” due to the diffusion technique [18]. Other MRI techniques applied in mice or rodents have ambitioned to better identify the fibrotic nature of a stricture, such as dynamic contrast enhanced MRI studies (with injection of Gd-DTPA) [19] or non-contrast MRI magnetization transfer and T2-weighted signal intensity ratio (comparing bowel wall intensity at the area of greatest thickening, to paraspinous muscle signal intensity) [20]. CT-colonoscopy, an imaging technique applied to adult IBD patients, is seldom used in pediatric patients due to concerns on radiation exposure in children [21]. Thus we chose not to use this technique on pre-pubertal animals.

Moving on to the second objective of our study, feeding rats exclusively with MODULEN IBD® during study protocol did not have an anti-inflammatory effect based on histological scores and MRI images.

Compared to the TNBS group, rats in the MODULEN group had significant growth failure starting within 6 days after first dose of TNBS, without subsequent catch-up growth. This growth failure could be explained by the colitis in itself, although nutritional factors could have amplified the weight loss. Indeed, although the rat breeding diet fed to the TNBS group has a high protein content, MODULEN IBD® is on the contrary low in protein and high in fat, with 42% energy from fat, 44% from carbohydrates and 14% from protein. The growth curve of the MODULEN IBD® group is very similar to that of a group of rats fed a protein-restricted diet (12% crude protein), in a model studying compensatory growth [22].

Protein restriction alone does not seem to explain why the severity of colitis in the MODULEN group was not less than in TNBS group. The majority of rats from the MODULEN group had moderate to severe inflammation and coexisting fibrosis on histology. MRI showed signs of intense inflammation with increased thickness and signs such as ulcers and a mucosal flap that were not seen in the TNBS group, but did not show significant stenosis. This would tend to suggest a pro-inflammatory effect in the MODULEN group.

Once again, nutritional aspects of the diet could explain in part these observations. Xue Li et al*.* showed that a high fat diet (60% energy from fat compared to 10% in controls) given for 4 weeks before induction of TNBS colitis in C57BL/6 mice increased severity of weight loss and histological damage, and increased pro inflammatory cytokines [23]. They showed that high fat diet caused an increase in oxidative stress, tight junction dysfunction and increased mucosal permeability.

We chose to use MODULEN IBD® in its original form and not as a supplementation to the rodents’ standard diet as other teams have done [9, 24] in order to best reproduce the effect of an exclusive enteral nutrition by MODULEN IBD® as recommended in pediatric IBD induction therapy. We also chose not to administer the MODULEN IBD® by oral gavage since the model was to be the least invasive possible. We had observed in a previous study of MODULEN treatment in acute TNBS colitis that the rats ate the powder and did not develop growth failure (tail length) over a period of 10 days.

Admitting the alternative hypothesis that difference in macronutrient composition is not significant in explaining the increased severity of colitis in the MODULEN group, another explanation would be that a component of the diet had a pro-inflammatory effect. A characteristic of MODULEN IBD® is its enrichment in TGF-beta 2 (> 24 ppm) [25]. TGF- β has several isoforms. TGF-β1 is the prominent cytokine involved in fibrotic procedures in multiple organs, through a signaling cascade leading to differentiation of fibroblasts to a myofibroblast phenotype which in turn causes collagen deposition in the extracellular matrix. TGF-β2 is classically associated with an anti-inflammatory effect. However there have been recent reports of increased TGF-β2 levels in colon strictures in a rat model of 2,4,6-trinitrobenzene sulfonic acid (TNBS) colitis [26]. In our study, not only were there fewer strictures in the MODULEN group, but also a trend to a decrease in all fibrosis markers compared to controls, although only alpha-SMA decrease was significant.

As in other animal models of intestinal fibrosis, we used histology as the gold standard for confirming fibrosis and therefore validity of the chronic model [27, 28]. One reason we did not observe an increase in “classical” fibrosis markers such as CTGF, alpha-SMA and COL1A1 could be the location of the sample. Indeed, in patients with stricturing ileal Crohn’s disease there has been shown an increase in alpha-SMA and COL I–III expression only in stenotic tract, and not in non-involved ileum [29].

However, considerations such as timing of analyses are also paramount, due to the dynamic nature of the fibrotic process. Briefly, fibrosis in the intestine is based on increased extracellular matrix production by activated myofibroblasts, and decreased degradation of these extracellular matrix components [30–32]. Myofibroblasts can originate from fibroblasts and from multiple other cellular sources, via epithelial to mesenchymal transition (EMT- and endothelial to mesenchymal transition (EndoMT). Many growth factors such as TGF-beta and CTGF are implicated in activation of mesenchymal cells, either alone or by cooperative interaction [33]. Upon activation, myofibroblasts express alpha-SMA, which is commonly used in studies as a marker of activated myofibroblast presence. Once activated, myofibrobasts produce extracellular matrix components according to a certain temporality. For example, Zhu et al*.*, in a chronic (5-doses of TNBS) colitis model in adult Sprague–Dawley rats showed increased expression of COL1A and COL3 mRNA as soon as the 2nd week of colitis, and collagen deposition starting at 3rd week [34]. During the final resolution of fibrosis phase, myofibroblasts are reduced by apoptosis [35].

When applying these theoretical considerations to our model, we can hypothesize that the timing at which the colons were harvested coincided with the beginning of resolution of colon fibrosis. Indeed, histology showed submucosal fibrosis, but the markers of myofibroblast activation (CTGF and COL1A1) were not increased compared to controls suggesting the myofibroblasts were no longer activated. On the contrary, the decrease of alpha-SMA in the MODULEN group could be interpreted as a sign of myofibroblast apoptosis and possibly, as an anti-fibrotic effect of MODULEN IBD®. This would need to be confirmed by serial analyses, with a longer follow-up after last dose of TNBS in order to confirm accelerated resolution of fibrosis in the MODULEN group compared to the TNBS group.

We acknowledge that our study has several limitations, in particular that power was limited by a higher than expected animal mortality. In addition, a longer follow-up with serried analyses would have been necessary in order to validate our hypothesis of an accelerated resolution of fibrosis in the MODULEN group. The strengths of the study were to have taken into account the particularities of pre-pubertal vs adult models. It is the first study showing that MRC is feasible in pre-pubertal rats and correlated to histology. It also gives insight into future studies of nutritional compounds in intestinal fibrosis.

## Conclusions

This model of chronic colitis in pre-pubertal rats caused significant inflammation and fibrosis based on histology and MR-colonography, and could be suitable for future therapeutic studies on developing animals. Absence of growth failure was probably explained by nutritional supplementation effect of rodent breeding diet. MODULEN IBD® administered a sole source of nutrition did not ameliorate colitis, although there was an interesting decrease in pro-fibrotic markers that would require further study.

## Supplementary Information

**Additional file 1**. Completed ARRIVE Guidelines checklist.

## Data Availability

All data generated or analysed during this study are included in this published article as additional file (Additional file 1).

## Abbreviations

TNBS: 2,4,6-Trinitrobenzene sulfonic acid
PBS: Phosphate-buffered saline
COL1A1: Alpha-1 type 1 collagen
CTGF: Connective tissue growth factor
GAPDH: Glyceraldehyde 3-phosphate dehydrogenase
α-SMA: Alpha-smooth muscle actin
COX-2: Cyclooxygenase 2
MR-colonography: Magnetic resonance colonography
RARE: Rapid acquisition with relaxation enhancement
FOV: Field of view
FLASH: Fast low angle shot
